# Effect of shade and thickness on the microhardness of resin-based composite specimens at different points considering curing light beam’s inhomogeneity

**DOI:** 10.1186/s12903-024-04647-2

**Published:** 2024-07-30

**Authors:** Lan Wang, Hyemin Ku, Dohyun Kim, Sung-Ho Park

**Affiliations:** Department of Conservative Dentistry and Oral Science Research Center, Yonsei University College of Dentistry, 50-1 Yonsei-ro, Seodaemun-gu, Seoul, 03722 Republic of Korea

**Keywords:** Beam inhomogeneity, Light curing unit, Microhardness, Resin-based composite, Thickness and shade

## Abstract

**Background:**

Recent studies have reported the inhomogeneity in the light emitted by dental light-curing units (LCUs). It is essential to understand how this uneven light distribution affects the physical properties of resin-based composites (RBCs) at various points across their surfaces. This study aimed to evaluate the effect of LCU beam’s inhomogeneity on the microhardness of RBCs with different shades and thicknesses.

**Methods:**

Four body (A1B, A2B, A3B, and A4B), one dentin (A3D), and one enamel shade (A3E) of RBC (Filtek Z350 XT) were examined. The specimens were fabricated in four thicknesses (1, 2, 3, and 4 mm) and subjected to a 40-second light-curing. Vickers microhardness testing was performed at the center point, and 3 mm left and right from the center at the bottom surface of each sample. The LCU beam profile was characterized using a beam profiler, while irradiance after specimen passage was measured using a spectrometer. One-way analysis of variance (ANOVA) and Tukey’s post-hoc tests were used to analyze the effects of shades and thicknesses on irradiance and microhardness, respectively. One-way repeated-measures ANOVA was used to compare the microhardness across different points. Pearson’s correlation analysis examined the relationship between irradiance and microhardness.

**Results:**

The beam profile of LCU revealed inhomogeneous light distribution. Light irradiance was decreased with both the increase in thickness and darker shade of the specimens (*p* < 0.05). Microhardness was found to decline with an increase in sample thickness (*p* < 0.05), and was consistently higher at the center point compared to the periphery, particularly in thicker (3 and 4 mm) and darker shades (A3B, A4B, and A3D). A positive correlation was found between the irradiance and microhardness across all evaluated points (*p* < 0.05).

**Conclusions:**

Inhomogeneous light emission from LCU significantly influences the microhardness of RBC samples, depending on the thicknesses and shades. The findings underline the importance of considering LCU beam inhomogeneity in clinical settings to ensure optimal polymerization of RBC.

## Background

Resin-based composites (RBCs) have been widely used in modern clinical dentistry for the esthetic direct restoration of teeth with dental caries or fractures. To ensure optimal polymerization of RBCs, the light-curing unit (LCU) should be able to deliver the required radiant exposure and spectral range, while simultaneously preventing oral tissue damage due to excessive heat [1].

The light outputs of LCUs can be measured with dental radiometers. Current standards such as the International Organization for Standardization (ISO) 6050, which dictate the calculation of radiant exitance (irradiance), presuppose a uniform distribution of power and spectral output from the light tip—a simplification represented by a single averaged value. Similarly, ISO 11,405 and ISO 4049, which guide bond strength testing and depth of cure assessments, respectively, assume uniform light distribution across the specimen’s surface [2]. However, several studies suggests that the emission from many dental LCU tips is neither radially symmetric nor homogeneous [3]. Consequently, relying on averaged irradiance and spectral measurements may not accurately reflect the true influence of LCU light output on the polymerization of RBCs. Therefore, a closer examination of the specific interactions between these inhomogeneous light outputs and RBC surfaces is needed.

While existing research primarily reveals the correlation between the heterogeneity of light emission and the microhardness of RBCs [4–6], Ko et al. have reported that environmental factors which reduces the light intensity, such as neutral density filters or the aperture sizes in charge-coupled device (CCD) cameras, can modify the LCU’s beam profile and the effects of the inhomogeneous beam on the RBCs [7]. They found that inhomogeneity in surface microhardness was typically observed in some RBCs when bulk-cured to a depth of 4 mm, whereas this finding was not replicated in bulk-cure type RBCs. Despite this, studies which provided comprehensive data examining the impact of LCU beam inhomogeneity on RBCs of varying shades and opacities remain scarce.

The polymerization of RBCs is influenced by various factors, including shade variation [8], increment thickness, cavity diameter and location [9], LCU employed [10], intensity and duration of light exposure [11], distance of the light-curing tip from the RBC surface, the substrate through which light-curing is performed, the type of filler, and the temperature during light-curing [11]. Go et al. noted that the polymerization of resin cements under ceramic specimens became inconsistent at ceramic thicknesses of 2 mm or greater, and the discrepancy was more evident as ceramic thickness increased [12]. This inhomogeneity was directly related to a rapid drop in the LCU beam’s irradiance passing through the ceramics with increased thickness. However, whether this effect is also present in light-curing of direct RBC has not been reported yet.

Therefore, this study aimed to investigate the influence of LCU beam inhomogeneity on the polymerization of RBCs, considering different composite thicknesses and shades. Additionally, the study evaluated the impact of varying irradiance on the uniformity of RBC polymerization. The null hypotheses were as follows:

1. There is no significant difference in irradiance among RBCs of different thicknesses and shades.
2. There is no significant difference in surface microhardness across RBC thicknesses.
3. There is no significant difference in surface microhardness among different RBC shades.
4. There is no significant difference in surface microhardness at different points on the surface of RBCs concurrently exposed to the LCU beam.

## Methods

### Material preparation

For this study, six different shades of a nanofill RBC (Filtek Z350 XT; 3 M ESPE, St. Paul, MN, USA) were used, including four body shades (A1B, A2B, A3B, and A4B), one dentin shade (A3D), and one enamel shade (A3E). Light activation was conducted using a light emitting diode LCU (Elipar DeepCure-S; 3 M ESPE, St. Paul, MN, USA) with a functional diameter of 9 mm. Radiant power of the light was measured using a spectrometer (USB4000; Ocean Optics, Dunedin, FL, USA) connected to an integration sphere (Labsphere; Ocean Optics, Dunedin, FL, USA) with an inner diameter of 6 inches.

Teflon moulds were fabricated to thicknesses of 1, 2, 3, and 4 mm, each with a central hole of 10 mm diameter. RBC samples were placed into the holes in Teflon molds, leveled with a 1 mm-thick glass plate, and then bulk-cured for 40 s (seconds) using the LCU. Ten specimens for each of the 24 groups—six shades and four thicknesses, a total of 240 specimens were prepared, and stored under light-proof conditions for seven days before testing for irradiance and microhardness. For A3D shade samples at 4 mm thickness, the bottom surfaces were not adequately polymerized even after the light curing for 40 s. Any uncured RBC was removed, resulting in a specimen thickness of approximately 3.3 mm.

### LCU beam profile measurement

The beam profile measurements followed the methodology of Ko et al. [7]. An LCU beam’s irradiance distribution was recorded using a laser beam profiler (BGS-LT665; Ophir Spiricon, Logan, UT, USA) and a CCD camera lens (LT665; Ophir Optronics, Jerusalem, Israel) with a 25 mm focal length and an f/4 aperture diaphragm. The LCU was placed over a holographic diffuser with a 60° diffusing angle (Edmund Optics, Barrington, NJ, USA), and a neutral-density filter with a 4.0 optical density (OD) value (Edmund Optics, Barrington, NJ, USA) was positioned in front of the camera lens. The distance between the CCD camera lens and the holographic diffuser was set at 32 cm.

### Measurement of irradiance after passing through RBC specimens

Five specimens from each group were randomly selected and subjected to irradiance testing. Each specimen was affixed to the LCU tip using black adhesive paper and the LCU was activated. The transmitted light was measured using a cosine corrector (CC-3-UV-2; Ocean Optics, Dunedin, FL, USA) coupled to a spectrometer (Flame-S; Ocean Optics, Dunedin, FL, USA). The irradiance (mW/cm^2^) was calculated based on the light passing through by dividing the area of optical fiber with a diameter of 3.9 mm.

### Surface microhardness test

The bottom surfaces of the remaining five specimens per group were finished with 1200-grit silicon carbide (SiC) paper to create smooth polished surfaces for surface microhardness testing. After polishing, thickness verification was conducted using a digital caliper with ± 0.1 mm precision. Vickers microhardness (HV) was measured at three points; the center, and 3 mm to the left and right of the center, using a hardness testing machine (MMT-X; Matsuzawa, Akita, Japan) under a load of 980.7 mN for 10 s at each point.

### Statistical analysis

The effects of RBC shade and thickness on irradiance were analyzed respectively using one-way analysis of variance (ANOVA) and Tukey’s post hoc tests. A similar analysis was conducted for the effects of RBC shade and thickness on microhardness using one-way ANOVA as well, and repeated-measures one-way ANOVA with the least significant difference test was used to examine the difference of microhardness across measurement points. Pearson’s correlation coefficients were calculated to assess the relationship between irradiance and surface microhardness at each point. All statistical analyses were performed under a 95% confidence level using the SPSS Statistics 26 (IBM Corp, Armonk, NY, USA) software program.

## Results

### LCU beam profile

**Fig. 1:**
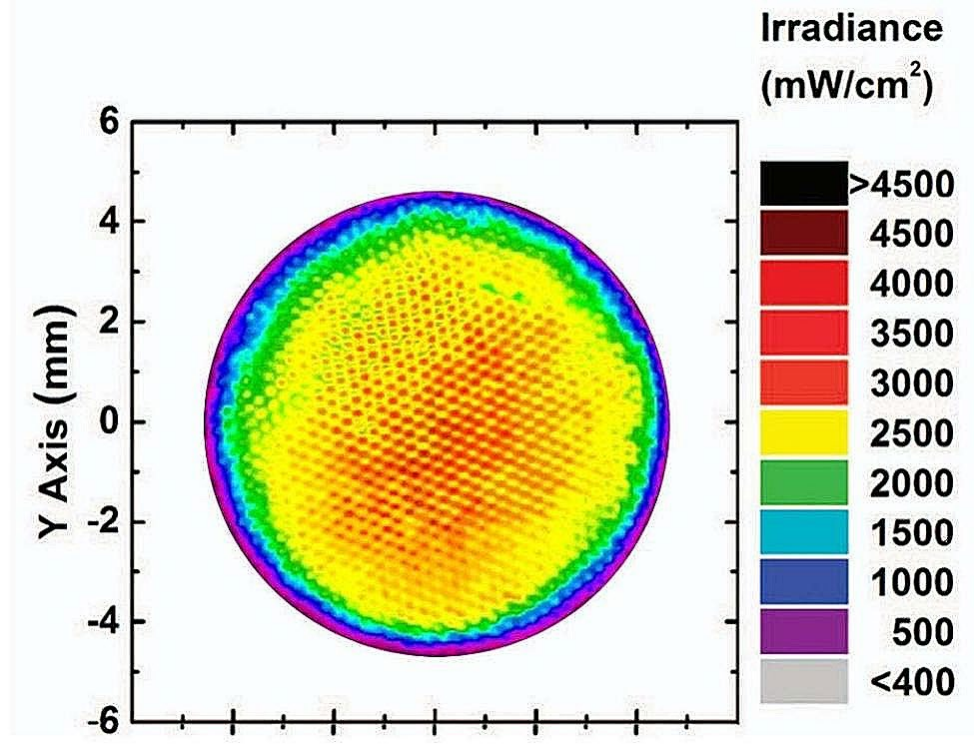
Representative beam profile image of the light-curing unit (Elipar DeepCure-S; 3 M-ESPE, St. Paul, MN, US)

The radiant emittance of the LCU was calculated as 1,015 mW/cm^2^, reflecting the diameter of functional light guide of the LCU was 9 mm. The inhomogeneous distribution of the LCU beam profile was evident, with energy progressively diminishing from the center towards the periphery (Fig. 1). Most of the light output was concentrated in a circular area about 6 mm in diameter near the center of the light tip. The irradiance peaks were associated with the light emitting diode (LED) chip locations or reflections from the reflectors within the LCU body.

### Irradiance after passing through RBC specimen

The analysis revealed significant differences in irradiances among different shades and thicknesses (*p* < 0.001 for both) (Table 1). In the post-hoc test, irradiance values for thicknesses were sequentially higher for 1 mm than 2 mm, 3 mm, and 4 mm specimens, with the exception of the A3D shade where 3 mm and 4 mm thicknesses did not differ significantly. There was a notable decrease in irradiance with increasing specimen thicknesses. Regarding shade-specific irradiance, the post-hoc test ranked the values from highest to lowest as follows: A1B > A3E > A2B > A3B > A4B > A3D. Darker and more opaque shades of RBC corresponded with lower light transmission.

**Table 1:** Mean and standard deviation of light irradiance (mW/cm^2^) of the light-curing unit (LCU) (Elipar DeepCure-S; 3 M-ESPE, St. Paul, MN, US) after the LCU Beam passed through resin-based composite specimens with different shades and thicknesses

	Shade					
Thickness	A1B	A2B	A3B	A4B	A3D	A3E
1 mm	203 ± 14.0^Aa^	171 ± 11.1^Ab^	144 ± 12.6^Ac^	121 ± 11.4^Ad^	57 ± 8.5^Ae^	171 ± 11.5^Ab^
2 mm	98 ± 5.7^Ba^	73 ± 8.7^Bc^	55 ± 8.2^Bd^	36 ± 4.9^Be^	15 ± 3.3^Bf^	90 ± 8.8^Bb^
3 mm	36 ± 4.4^Ca^	29 ± 7.5^Cb^	23 ± 8.2^Cc^	11 ± 4.3^Cd^	2 ± 0.7^Ce^	34 ± 5.1^Ca^
4 mm	17 ± 1.4^Da^	12 ± 4.0^Db^	8 ± 1.3^Dc^	3 ± 1.1^Dd^	^*^1 ± 0.3^Ce^	16 ± 2.1^Da^

Different uppercase letters denote significant differences among different thicknesses within each shade (columns), and different lowercase letters denote significant differences among different shades within each thickness (rows) (*p* < 0.05)

^*^The bottom surfaces of the 4 mm-thickness A3D specimens were not adequately polymerized even after light curing for 40 s. The measurement was taken after removing the unpolymerized RBC material, resulting in a specimen thickness of approximately 3.3 mm.

### Surface microhardness

**Fig. 2:**
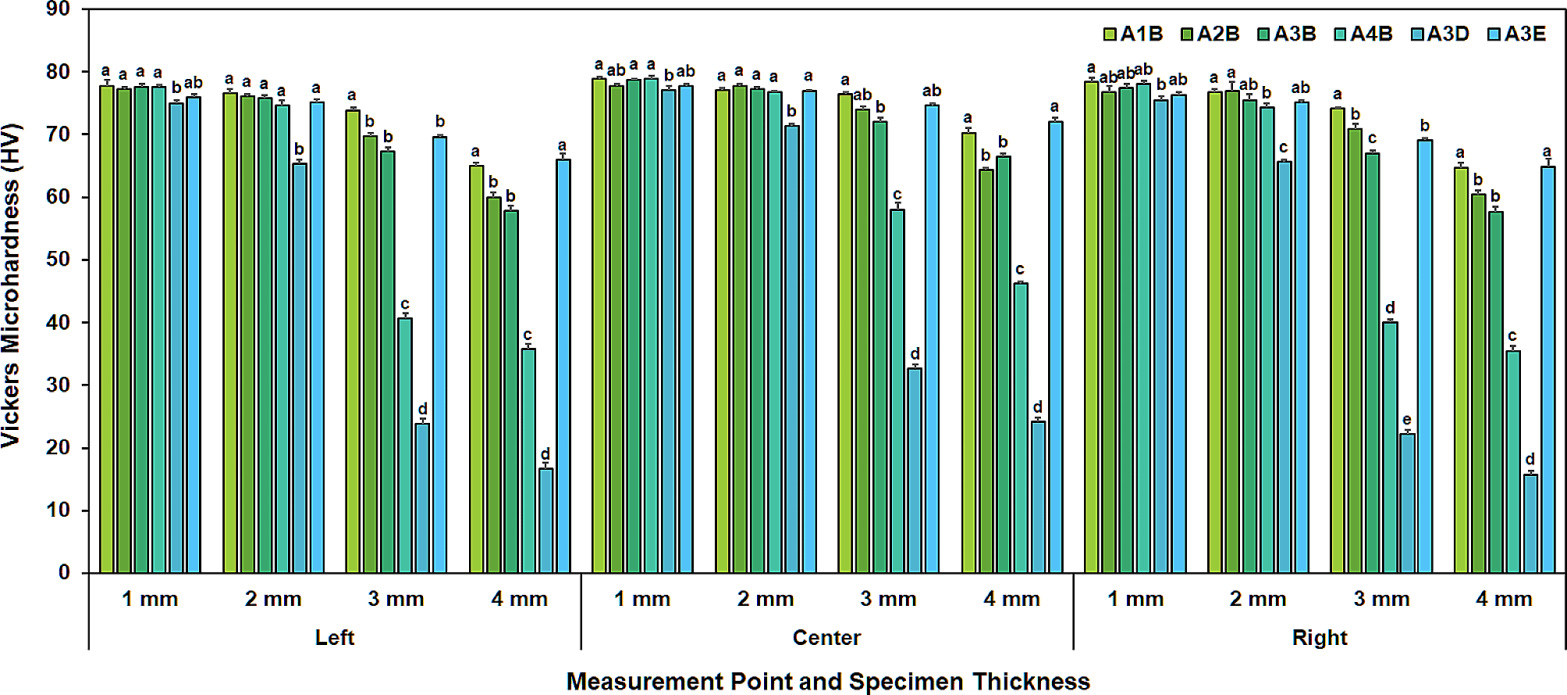
Mean surface microhardness (HV) of the resin-based composite (RBC) specimens at each measuring point among different shades and thicknesses. Different lowercase letters denote statistical differences among different RBC shades within same thickness and measurement point (*p* < 0.05)

Significant differences in surface microhardness among thicknesses within the same shade group were detected (*p* < 0.05) (Tables 2 and Fig. 2). With the exception of A1B center, A4B left and right, and A3D center, left, and right, there were no significant differences in HV values between 1 mm and 2 mm thick specimens. For A1B center, A4B left and right, and A3D center, left, and right, the 1 mm thick specimens showed significantly higher HV values than the 2 mm specimens (*p* < 0.05). In all specimens except A1B center, HV values significantly decreased as thickness increased from 2 mm to 3, and 4 mm (*p* < 0.05). For A1B center, there was no significant difference in HV values between 2 mm and 3 mm thicknesses.

A significant effect of RBC shade on surface microhardness was apparent across all thicknesses and measurement points (*p* < 0.05). For the 1- and 2-mm specimens, shades showed no significant differences in the post-hoc tests except for A3D, which was significantly lower in microhardness compared to other shades (*p* < 0.05). In the 3-mm thickness category, HV levels were arranged as A1B > A3E, A2B > A3B > A4B > A3D, and for the 4-mm thickness group as A1B, A3E > A2B, and A3B > A4B > A3D (*p* < 0.05). The microhardness decreased from center to periphery, with significant differences observed across measuring points (*p* < 0.05), particularly pronounced in thicker (3 and 4 mm), darker (A3B and A4B), and more opaque (A3D) specimens.

**Table 2:** Mean and standard deviation of surface microhardness (HV) of resin-based composite specimens at each measurement point among different shades and thicknesses

Shade	Measurement point	Thickness			
		1 mm	2 mm	3 mm	4 mm
A1B	left	77.8 ± 1.7^Aa^	76.6 ± 1.3^Aa^	73.9 ± 0.7 ^Bb^	65.0 ± 1.1 ^Cb^
	center	78.9 ± 0.7 ^Aa^	77.1 ± 0.6 ^Ba^	76.4 ± 0.7 ^Ba^	70.3 ± 1.5 ^Ca^
	right	78.4 ± 1.4 ^Aa^	76.7 ± 1.0 ^Aa^	74.1 ± 0.4 ^Bb^	64.7 ± 1.5 ^Cb^
A2B	left	77.2 ± 0.8 ^Aa^	76.1 ± 0.8 ^Ab^	69.7 ± 0.9 ^Bb^	60.0 ± 1.4 ^Cb^
	center	77.8 ± 0.6 ^Aa^	77.8 ± 0.6 ^Aa^	74.0 ± 1.0 ^Ba^	64.3 ± 0.7 ^Ca^
	right	76.7 ± 2.1 ^Aa^	76.9 ± 0.3 ^Aab^	70.9 ± 1.7 ^Bb^	60.4 ± 1.3 ^Cb^
A3B	left	77.6 ± 1.0 ^Aa^	75.8 ± 1.0 ^Aa^	67.3 ± 1.4 ^Bb^	57.8 ± 1.8 ^Cb^
	center	78.8 ± 0.3 ^Aa^	77.2 ± 0.8 ^Aa^	72.0 ± 1.3 ^Ba^	66.4 ± 1.2 ^Ca^
	right	77.4 ± 1.5 ^Aa^	75.5 ± 1.8 ^Aa^	67.0 ± 0.8 ^Bb^	57.7 ± 1.4 ^Cb^
A4B	left	77.5 ± 0.9 ^Aa^	74.6 ± 1.6 ^Bb^	40.6 ± 1.7 ^Cb^	35.7 ± 1.7 ^Db^
	center	78.9 ± 1.0 ^Aa^	76.8 ± 0.4 ^Aa^	58.0 ± 2.3 ^Ba^	46.2 ± 0.7 ^Ca^
	right	78.0 ± 1.2 ^Aa^	74.3 ± 1.4 ^Bb^	40.0 ± 1.0 ^Cb^	35.5 ± 1.4 ^Db^
A3D	left	75.0 ± 0.9 ^Ab^	65.3 ± 1.2 ^Bb^	23.8 ± 1.8 ^Cb^	^*^16.6 ± 2.0 ^Db^
	center	77.1 ± 1.4 ^Aa^	71.3 ± 0.8 ^Ba^	32.6 ± 1.5 ^Ca^	^*^24.1 ± 1.4 ^Da^
	right	75.5 ± 1.1 ^Aab^	65.6 ± 0.7 ^Bb^	22.2 ± 1.2 ^Cb^	^*^15.7 ± 1.2 ^Db^
A3E	left	75.9 ± 1.0 ^Ab^	75.1 ± 1.2 ^Ab^	69.6 ± 0.6 ^Bb^	66.0 ± 1.9 ^Cb^
	center	77.7 ± 0.6 ^Aa^	77.0 ± 0.3 ^Aa^	74.6 ± 0.7 ^Ba^	72.1 ± 1.3 ^Ca^
	right	76.3 ± 0.8 ^Ab^	75.1 ± 0.6 ^Ab^	69.1 ± 0.6 ^Bb^	64.9 ± 2.4 ^Cb^

Different uppercase letters denote significant differences among different thicknesses within each shade and measurement point (rows), and different lowercase letters denote significant differences among different measurement points within each shade and thickness (*p* < 0.05)

^*^The bottom surfaces of the 4 mm-thickness A3D specimens were not adequately polymerized even after light curing for 40 s. The measurement was taken after removing the unpolymerized RBC material, resulting in a specimen thickness of approximately 3.3 mm

### Correlation between irradiance and microhardness

**Fig. 3:**
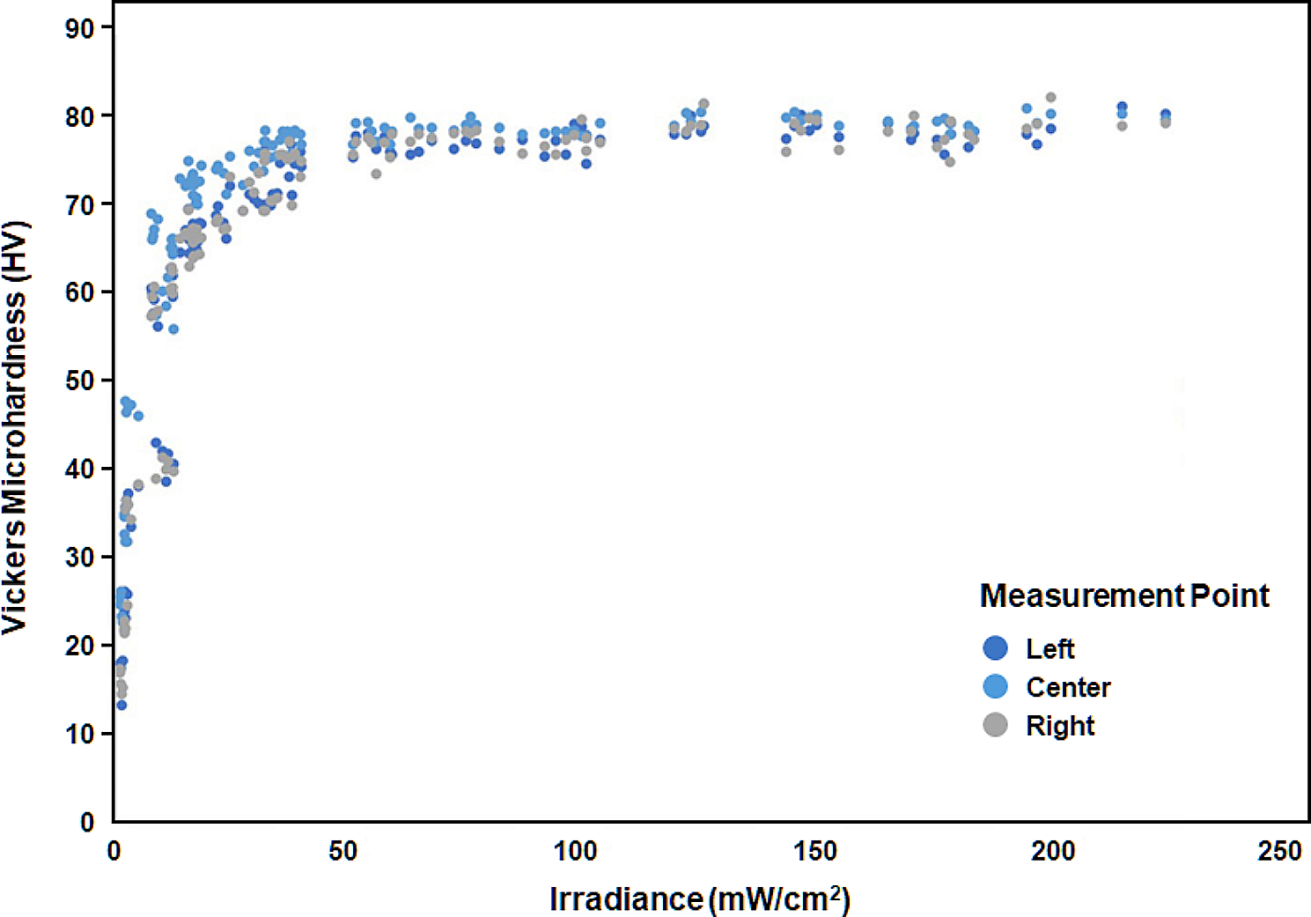
Correlation between light irradiance and surface microhardness (HV) of the resin-based composite specimens

A fair positive correlation was found between irradiances and surface microhardness at the center (*r* = 0.54, *p* < 0.001), and moderately strong positive correlations were found at the left (*r* = 0.60, *p* < 0.001), and right (*r* = 0.60, *p* < 0.001) measurement points (Fig. 3)

## Discussion

The findings of this study demonstrate a significant decrease in irradiance with increased RBC specimen thickness across all shades except between 3 and 4 mm A3D shade specimens, which is consistent with the results of a previous study [13]. This observation led to the rejection of the first null hypothesis. The irradiance was lower with increasing shade number among the four body shades (A1B, A2B, A3B, and A4B), and increased opacity resulted in a significant decrease in irradiance among the A3 shade groups (A3E, A3B, and A3D).

According to a previous study, inner composition and structure of the examined material could affect light transmission [14]. Light transmission through RBCs comprised of diffuse and straight-line transmissions, resulting from light scattering at the surface of filler particles and matrices. As the thickness of the resin composite increases, more scattering and refraction could occur within the material [15, 16]. In a similar context, several studies have recommended a longer exposure time for greater thicknesses, as the degree of conversion decreases when material thickness increases [17, 18]. In this study, the specimen thickness was up to 4 mm, therefore, we used an exposure time of 40 s instead of the manufacturer’s recommended 20 s.

In this experiment, a 1 mm thick glass slide was used to ensure the composite resin specimens were made with a uniform thickness. ISO 4049 recommends a minimum light intensity of 300 mW/cm² for composite resin polymerization experiments, while most clinical guidelines suggest 600–1000 mW/cm² to account for distance and light loss. Additionally, the decrease in light intensity due to distance is reported to be less than 10% within the first 1 mm from the curing tip [19]. Considering the light intensity of the curing unit used in the experiment was 1, 015 mW/cm², it is deemed sufficient for the experiment.

We polished the specimen surface with 1200-grit SiC paper to achieve a smooth surface similar to that in actual clinical settings. Specimens that are not polished may have their surface resin matrix intact, which can result in a lower measured microhardness than actual [20, 21]. SiC paper #1200 has a grit size of 15.3 μm, similar to a Sof-Lex fine polishing disc (14 μm, 3 M ESPE, St Paul, MN, USA).

Surface microhardness was found to decrease with increasing thickness beyond 2 mm, except for A3D (left and center) and A3B (left) shades which exhibited this trend starting from a thickness of 1 mm. Based on these results, the second null hypothesis was partially rejected. Bouschlicher et al. suggested that to define the depth of cure based on top and bottom hardness measurements, the ratio of bottom/top hardness should be given an arbitrary minimum value in order to consider the bottom surface as adequately cured [22]. Subsequently, numerous studies have adopted the 80% level of the maximum microhardness value as a measure of adequate polymerization in clinical settings [18, 23, 24]. In this study, the Vicker’s microhardness value in 1 mm is approximately 75. Assuming this is 100%, the microhardness corresponding to 80% becomes 60. There was no specimen with microhardness value under 60 in 2 mm thickness, but some were found in A4B and A3D specimens with 3 and 4 mm thicknesses. This may be related to the very low irradiance of these two shades in 3 and 4 mm-thick specimens. The result that the A3D specimen was only polymerized up to 3.3 mm even after 40 s of light curing supports this finding. These results were consistent with those of a previous study using same material (Filtek Z350 XT A3B shade), which reported that no discernible difference in microhardness was found between 1 mm and 2 mm-thickness specimens, and specimens with more than 3 mm of thicknesses failed to demonstrate adequate polymerization [7]. This may be attributed to the fact that light intensity was significantly diminished while passing the bulk of RBC specimens due to light scattering and absorptions, which could compromise polymerization efficiency.

The study also observed no substantial differences in microhardness between shades at 1 and 2 mm thickness, except in the A3D shade, which exhibited significantly lower microhardness at the left position in a 2 mm-thickness specimen. On the contrary, microhardness between different shades were more evident in 3 and 4 mm-thicknesses specimens, necessitating a partial rejection of the third null hypothesis. This is consistent with the results of previous studies, which reported that RBCs with lighter shades revealed higher microhardness and higher degree of polymerization than darker ones that require more curing time to improve microhardness [25, 26]. The shade and light transmittance of RBCs were affected by the light-scattering behavior caused by the filler shape, size, content, and the difference in the refractive indices between the filler particles and the resin matrix [27, 28]. Owing to the increasing intensity and opacity of shades, light transmission diminished when passing through them.

The microhardness of RBCs decreased from the center to the periphery, and this tendency was more evident in thick (3 and 4 mm), dark (A3B and A4B), and opaque (A3D) shades (Table 2). Therefore, the fourth null hypothesis was also partially rejected. At 1 mm thickness, no difference in the microhardness was found between the center, left, and right positions, except for A3D and A3E. In 2 mm thickness, only A1B, and A3B did not show differences between them. In 3 and 4 mm, significant differences in all shades were found between them. When there were differences in the microhardness between positions, it was always the same or higher in the center position than in the right and left positions. This means that the effect of the inhomogeneity of the LCU beam on the microhardness of RBCs becomes greater as the thickness increases and as the color becomes darker and opaque. These results are likely to be related to the irradiance of the LCU, which is affected by the depth or color tone of RBCs.

Positive correlations were found between the irradiances and microhardness values in the measuring points, and the correlation in the left and right (*r* = 0.60; moderately strong correlation) was higher than that in the center (*r* = 0.54; fair correlation) in all shades and thicknesses. This means that the microhardness of the periphery is more sensitive to irradiance than that of the center and is consistent with the findings of Go et al. [12]. They placed dual-cured or light-cured resin cements under ceramics of various thicknesses, activated the LCU over the ceramics, and measured the microhardness of the resin cements at 5 points from the center to the periphery for comparison. They found that resin cements under a 1-mm CAD-CAM block showed relatively uniform microhardness, whereas those under 2-mm, and 4-mm blocks showed inhomogeneous microhardness. They reported that the thicker the ceramics, the more inhomogeneous the LCU beam passing through it and resulted in inhomogeneous microhardness in resin cements under thick ceramics.

The image of the LCU’s beam profile showed that the LCU delivered high-output levels near the center of the light tip and the energy decreased toward the periphery, which informs the inhomogeneous irradiance distribution across the light tip. Ko et al. [7] reported that the attenuation factors such as the aperture of a camera lens or neutral-density filters affected the LCU’s beam profile. This study was conducted based on their methods.

In this study, a significant difference in microhardness between the center and the periphery of the specimens was observed for certain shades. However, for 1-mm and 2-mm specimens, the difference in values was not substantial, and all microhardness values were measured to be above 80% of the maximum value. In contrast, for 3-mm and 4-mm specimens, the difference in microhardness values between the center and periphery was more pronounced. In darker and more opaque shade specimens, results falling below 80% of the maximum microhardness were observed (e.g., 3-mm A4B, A3D shades and 4-mm A3B, A4B, A3D shades). According to the manufacturer’s instructions, the bottom surface of the Filtek Z350 XT specimen must be polymerized before more than 2 mm of the composite was built up. However, in this study, bulk filling was intentionally performed at 3 and 4 mm to find the correlation among the LCU’s beam profile, its irradiance, and microhardness in varying thicknesses and shades of RBCs. The problems presented in this experiment caused by the inhomogeneous LCU beam would not occur if incremental light curing had been done every 2 mm.

In this study, we used only one LCU, the Elipar DeepCure-S. Previous studies have shown that the pattern of inhomogeneity varies among different LCUs, which may influence the results [7]. Price et al. conducted a similar study using a different LCU (Bluephase Style; Ivoclar Vivadent, Amherst, NY, USA) which demonstrated a strong positive correlation between the irradiance values corresponding to the beam profile distribution and the microhardness of the RBC specimens at the corresponding locations [4]. Their methodology involved creating a detailed microhardness map by dividing the specimen into 45 sections and analyzed the correlation between irradiance and microhardness across different areas. The Bluephase Style exhibits an asymmetrical beam profile due to its design, where two out of three light sources emit wavelengths targeting camphorquinone. In contrast, the Elipar DeepCure-S demonstrates a relatively more symmetrical beam profile, resembling the growth rings of a tree, with high irradiance at the center that gradually decreases towards the periphery. Considering these factors, we employed a simplified approach by measuring microhardness at the center and at points 3 mm to the left and right. Further research is needed using LCUs with various inhomogeneity patterns, as well as more precise and granular measurements and analyses.

## Conclusions

This study confirms that the inhomogeneity of light emitted by LCUs influences the polymerization quality of RBCs, significantly impacting surface microhardness across various shades and thicknesses. The correlation between the irradiance of LCU and the microhardness of RBCs reinforces the importance of standardized and constituent light exposure to ensure effective curing in clinical dentistry. It also suggests that deviations from recommended incremental filling techniques may lead to suboptimal hardness, and consequently, the longevity of RBC restorations.

## Data Availability

The datasets used and/or analysed during the current study are available from the corresponding author on reasonable request.

## Abbreviations

ANOVA: Analysis of variance
CCD: Charge-coupled device
HV: Vickers microhardness
ISO: International Organization for Standardization
LCU: Light-curing unit
LED: Light emitting diode
OD: Optical density
RBC: Resin based composite
s: Second
SiC: Silicon carbide
